# Factors associated with concurrent wasting and stunting among children 6–59 months in Karamoja, Uganda

**DOI:** 10.1111/mcn.13074

**Published:** 2020-08-23

**Authors:** Gloria Adobea Odei Obeng‐Amoako, Charles Amnon Sunday Karamagi, Joanita Nangendo, Jaffer Okiring, Yerusa Kiirya, Richmond Aryeetey, Ezekial Mupere, Mark Myatt, André Briend, Joan Nakayaga Kalyango, Henry Wamani

**Affiliations:** ^1^ Clinical Epidemiology Unit, School of Medicine, College of Health Sciences Makerere University Kampala Uganda; ^2^ Department of Paediatrics and Child Health, College of Health Sciences Makerere University Kampala Uganda; ^3^ School of Public Health University of Ghana Accra Ghana; ^4^ Brixton Health Llawryglyn Powys, Wales UK; ^5^ School of Medicine, Centre for Child Health Research University of Tampere Tampere Finland; ^6^ Department of Nutrition, Exercise and Sports University of Copenhagen Copenhagen Denmark; ^7^ Department of Pharmacy, College of Health Sciences Makerere University Kampala Uganda; ^8^ Department of Community Health and Behavioural Sciences, School of Public Health, College of Health Sciences Makerere University Kampala Uganda

**Keywords:** concurrent wasting and stunting, factors associated with WaSt, stunting, Uganda, wasting

## Abstract

Children with concurrent wasting and stunting (WaSt) and children with severe wasting have a similar risk of death. Existing evidence shows that wasting and stunting share similar causal pathways, but evidence on correlates of WaSt remains limited. Research on correlates of WaSt is needed to inform prevention strategies. We investigated the factors associated with WaSt in children 6–59 months in Karamoja Region, Uganda.

We examined data for 33,054 children aged 6–59 months using June 2015 to July 2018 Food Security and Nutrition Assessment in Karamoja. We defined WaSt as being concurrently wasted (weight‐for‐height z‐scores <−2.0) and stunted (height‐for‐age z‐score <−2.0). We conducted multivariate mixed‐effect logistic regression to assess factors associated with WaSt. Statistical significance was set at *p* < 0.05.

In multivariate analysis, being male (adjusted odds ratio [aOR] = 1.79; 95% confidence interval [CI] [1.60–2.00]), aged 12–23 months (aOR = 2.25; 95% CI [1.85–2.74]), 36–47 months (aOR = 0.65; 95% CI [0.50–0.84]) and 48–59 months (aOR = 0.71; 95% CI [0.54–0.93]) were associated with WaSt. In addition, acute respiratory infection (aOR = 1.30; 95% CI [1.15–1.48]), diarrhoea (aOR = 1.25; 95% CI [1.06–1.48]) and malaria/fever (aOR = 0.83; 95% CI [0.73–0.96]) episodes were associated with WaSt. WaSt was significantly associated with maternal underweight (body mass index <18.5 kg/m^2^), short stature (height <160 cm), low mid‐upper arm circumference (MUAC <23 cm) and having ≥4 live‐births. WaSt was prevalent in households without livestock (aOR = 1.30; 95% CI [1.13–1.59]).

Preventing the occurrence of WaSt through pragmatic and joint approaches are recommended. Future prospective studies on risk factors of WaSt to inform effective prevention strategies are recommended.

**Key messages**

- Concurrent wasting and stunting (WaSt) was significantly associated with child's age, child's sex, acute respiratory infection (ARI) and diarrhoea episodes especially among children of caregivers with low body mass index (BMI), short stature, low mid‐upper arm circumference (MUAC), ≥4 live‐births and living in households without livestock
- Existing and new programmes designed for wasting and stunting reduction should be deliberate in addressing both conditions, simultaneously, especially in high burden settings
- Studies on how integrated packages involving nutrition‐sensitive and nutrition‐specific interventions can be targeted at preventing child undernutrition are needed. Further studies on risk factors of WaSt are recommended

## 1 INTRODUCTION

Wasting and stunting are the commonest forms of undernutrition in children under 5 years. Annually, one‐third of all deaths in children under 5 years are attributed to undernutrition (Bhutta et al., 2013). The World Health Assembly (WHA) targets to reduce the global prevalence of wasting from 7.8% to 5%, and the 155 million stunted children by 40% by 2025 (World Health Organization, 2014). Among children aged under 5 years residing in Africa, 6.4% are wasted and 29% are stunted. The prevalence of wasting is 5.3% in Eastern Africa (United Nations Children's Fund [UNICEF], World Health Organization, & International Bank for Reconstruction and Development/The World Bank, 2020). Stunting prevalence is highest in Eastern Africa (34.5%) compared with the rest of Africa (UNICEF, World Health Organization, & International Bank for Reconstruction and Development/The World Bank, 2020). Childhood undernutrition is prevalent in Uganda; 4% and 29% of children under 5 years are wasted and stunted, respectively (Uganda Bureau of Statistics [UBOS] & ICF, 2018).

Emerging evidence indicates that wasting and stunting can co‐exist in some children (Garenne, Myatt, Khara, Dolan, & Briend, 2019; Khara, Mwangome, Ngari, & Dolan, 2018; McDonald et al., 2013; Myatt et al., 2018; Odei Obeng‐Amoako, Myatt et al., 2020; Odei Obeng‐Amoako, Wamani et al., 2020; Schoenbuchner et al., 2019). Due to the joint adverse effects of wasting and stunting, children with concurrent wasting and stunting (WaSt) have increased risk of death compared with being wasted alone or stunted alone (Garenne et al., 2019; McDonald et al., 2013; Myatt et al., 2018). The prevalence of WaSt is reported to range from 0% to 8.0% among children aged 6–59 months across 84 countries in Asia, Africa, Europe, Latin America and Oceania (Khara et al., 2018). Children with WaSt should be considered as a public health priority group for prevention and curative interventions.

Literature indicates that wasting and stunting share similar causal pathways and consequences (Briend, Khara, & Dolan, 2015; Harding, Aguayo, & Webb, 2018). A pooled odds ratio of 1.4 was estimated for being stunted if wasted and vice versa (Myatt et al., 2018). Some reports indicate that child age and sex, as well as exposure to food insecurity, are significantly associated with WaSt (Garenne et al., 2019; Myatt et al., 2018; Schoenbuchner et al., 2019). Other studies have focused on factors associated with multiple anthropometric deficits (Fentahun, Belachew, & Lachat, 2016; Hondru et al., 2019). According to Fentahun et al., factors associated with multiple anthropometric deficits are being female, a suboptimal dietary diversity and not being fed special foods during illness (Fentahun et al., 2016). Another study found that children who were concurrently wasted, stunted and underweight were two times more likely to have prolonged days of illness than those who were wasted or stunted (Hondru et al., 2019).

Understanding the correlates of WaSt is important for identifying and designing prevention and treatment strategies. Factors associatedwith the different categories of undernutrition in children have been widely studied. Undernutrition is attributed to complex and interconnected factors; poor dietary intake and morbidity are the proximal factors. Distal factors include poverty and socioeconomic status (UNICEF, 1990). However, few studies have focussed on factors associated with WaSt. We need more information on the correlates of WaSt to support integrated approaches to prevent and treat WaSt, and ultimately to avoid the occurrence of WaSt in children under 5 years. This study aimed to assess the factors associated with WaSt in children 6–59 months in Karamoja Region, Uganda.

## 2 METHODS

### 2.1 Data source

We conducted secondary data analysis of the June 2015 to July 2018 Food Security and Nutrition Assessment (FSNA) cross‐sectional survey datasets conducted in all the seven districts of the Karamoja Region in North‐Eastern Uganda. Data was pooled from seven FSNA datasets for the current study. These datasets have been described in detail elsewhere (Odei Obeng‐Amoako, Myatt, et al., 2020). The FSNA surveys were carried out by the Makerere University School of Public Health and the International Baby Food Action Network (IBFAN), Uganda, in collaboration with the United Nations (UN) Children's Fund (UNICEF), the UN Food and Agriculture Organization (FAO), the UN World Food Programme (WFP), the Department of Risk Reduction at the Office of the Prime Minister and the Ministry of Health, Uganda.

The FSNA protocol adapted the Standardized Monitoring and Assessment of Relief and Transitions (SMART) survey methodology (Golden et al., 2006). The FSNA employed a two‐stage cluster sampling approach. First, a probability proportional to size sampling procedure was used to select clusters from a list of parishes in each district. Secondly, households in each of the clusters were selected by systematic random sampling technique (UNICEF, Department for International Development [DFID], WFP, FAO, & IBFAN, 2018). The estimated number of children and households were selected from each of the clusters per the FSNA protocol (UNICEF, DFID, WFP, FAO, & IBFAN, 2018). FSNA adapted a standardized semistructured questionnaire for data collection by trained research assistants. The FSNA questionnaire consists of household characteristics, food security and nutrition modules. The household and food security modules were administered to household heads or adults present at the time of the interview while the mothers or caregivers (hereinafter referred as caregivers) of children under 5 years were interviewed using the nutrition module.

Weight and height measurements were collected from nonpregnant women with children aged 0–59 months in the household. Mid‐upper arm circumference (MUAC) was measured for both pregnant and nonpregnant women with children aged 0–59 months. Weight, height and MUAC measurements were collected from children aged 6–59 months found in the household. Recumbent length was measured for children <24 months old. Bilateral pitting oedema and haemoglobin levels among children aged 6–59 months were also examined. Child age in months was obtained from the child health cards or by maternal recall using a local event calendar.

### 2.2 Definition of childhood growth indices

We defined wasted as weight for height z‐score (WHZ) <−2.0, stunted as height for age z‐score (HAZ) <−2.0, and underweight as weight for age z‐score (WAZ) <−2.0 based on z‐scores of the 2006 World Health Organization (WHO) growth standards (WHO, 2006). Degrees of anthropometric deficits were defined as no deficit as z‐scores ≥−2; moderate deficits as z‐scores <−2 and ≥−3 and severe deficits as <−3 z‐scores. We defined acute malnutrition as wasting (WHZ <−2) and/or MUAC <12.5 cm; severe acute malnutrition (SAM) as severe wasting (WHZ <−3) and/or MUAC <11.5 cm and moderate acute malnutrition (MAM) as moderate wasting (WHZ ≥−3 to −2) and/or MUAC ≥11.5 cm and ≤12.5 cm (United Nations High Commissioner for Refugees [UNHCR] & WFP, 2011).

### 2.3 Study variables

The outcome of our study was WaSt among children aged 6–59 months. We defined WaSt as concurrently having WHZ <−2 and HAZ <−2 (Myatt et al., 2018). We explored a range of factors likely to be associated with WaSt in children aged 6–59 months in this analysis based on previous literature and on the UNICEF conceptual framework for under‐nutrition (UNICEF, 1990). These explanatory variables were classified as child level, maternal level (i.e., caregivers) and household characteristics as well as markers of the food security seasons during which the surveys were conducted.

### 2.4 Ethical considerations

We received approval to access the Karamoja FSNA datasets from the Office of the Prime Minister, Uganda. We used a de‐identified dataset for this analysis. We obtained a waiver of consent to use the FSNA datasets for this study from the Makerere University School of Medicine Higher Degrees Research and Ethics Committee. Ethical approval for the present analysis was granted by the Makerere University School of Medicine Higher Degrees Research and Ethics Committee and the Uganda National Council for Science and Technology.

#### 2.3.1 Child level factors

Child level factors included in the study were age in months, sex and morbidity. We categorized the age of children into 6–11, 12–23, 24–35, 36–47 and 48–59 months (Garenne et al., 2019). Childhood morbidity was reported by the caregivers. The presence of infectious disease was defined as ARI, malaria/fever, and diarrhoea episodes in the last 2 weeks before the survey.

#### 2.3.2 Maternal level factors

We considered age, the number of live‐births and nutritional status of caregivers. We categorized maternal age as 15–19, 20–29, 30–39 and >40 years (UBOS & ICF, 2018). Maternal educational level was described using the cumulative number of years of formal education attained. Educational level was categorized as no education, 1–7 years (primary) and ≥8 years (secondary and above). We also classified the number of live‐births of caregivers into 1–3, 4–6 and >7 number of live‐births. We categorized maternal body mass index (BMI) as normal (BMI = 18.5–24.9 kg/m^2^), underweight (BMI < 18.5 kg/m^2^), and overweight/obese (BMI ≥ 25 kg/m^2^) (UNHCR & WFP, 2011). Maternal height was categorized as tall (height ≥170 cm), moderately tall (height = 160–170 cm) and short (height <160 cm). Maternal MUAC signifying acute malnutrition was classified as low MUAC (MUAC <23 cm) and normal MUAC (MUAC ≥23 cm) (MoH Uganda & UNICEF, 2016; Ververs, Antierens, Sackl, Staderini, & Captier, 2013).

#### 2.3.3 Household factors and season of survey

The socioeconomic factors included in the analysis were household heads' sex, maternal and household heads' educational level, wealth index, livestock ownership, access to land for cultivation and season of survey. Similar to maternal educational level, we described household head's educational level as no education, 1–7 years (primary) and ≥8 years (secondary and above). We used principal component analysis to generate a wealth index based on household assets ownership (Rutstein & Staveteig, 2014). These household assets were bed, table, chair, mattress, radio/tape, cell phone, sewing machine, bicycle, automobile, motorcycle, television, axe, panga/machete, hoe, ox‐plough, water tank, seed and food stores (UNICEF, DFID, WFP, FAO, & IBFAN, 2018). We categorized the wealth index from the lowest to the highest as poorest, poor, middle, rich and the richest households using quintiles.

We used household livestock ownership and access to land for cultivation as an independent factor for wealth given that Karamoja is an agro‐pastoral setting. Household food consumption scores reflect the diversity and frequency of food items from the different food groups reportedly consumed over the previous 7 days. The food items were grouped into main staples, pulses, vegetables, fruits, meat/fish, milk, sugar and oil. The food consumption scores were weighted according to the relative nutritional value of the consumed food groups (International Dietary Data Expansion [INDDEX] Project, 2018). The thresholds of food consumption scores that were used to determine food consumption status of households were 0–21 (poor), 21.5–35 (borderline), >35 (acceptable) (INDDEX Project, 2018).

We evaluated the presence of a latrine in a household as a probable factor associated with WaSt. Sources of drinking water were defined as improved water sources (piped/tap, protected well/spring and borehole fitted with a hand‐pump) and unimproved water sources (surface water, river, dam, runoff, rainwater collected in a tank and water from open well/spring) (WHO & UNICEF, 2006). The association between food security seasons and the occurrence of WaSt was also examined. FSNA surveys were conducted in May/June for the lean (hunger or preharvest) and in November/December for the nonlean (postharvest) seasons.

#### 2.5 Study population

Children aged 6–59 months were included in the analysis. We used WHO flags for outliers; HAZ <−6 and HAZ >6, WAZ <−6 and WAZ >5, and WHZ <−5 and WHZ >5 were excluded as biologically implausible z‐scores values (WHO, 2009). Given that WaSt was the outcome variable, children with incomplete data on WHZ or HAZ or both were excluded from the study. Children with bilateral pitting oedema, height <45 and >120 cm and MUAC >20 cm were also excluded from the dataset (MoH Uganda & UNICEF, 2016). The maternal MUAC measurements which were out of range of upper quartile +3.0× interquartile range (IQR) and lower quartile −3.0× IQR were censored as outliers (Healy, Chambers, Cleveland, Kleiner, & Tukey, 1984).

#### 2.6 Data management and analysis

All statistical analyses were conducted using STATA 13.0. We also analysed anthropometric z‐scores per WHO growth standards in STATA (Leroy, 2011). We used descriptive statistics to summarize continuous variables using medians and IQRs and categorical variables using percentages.

We used multivariate mixed‐effect logistic regression to assess child level, maternal and household level, socioeconomic and seasons factors associated with WaSt. Mixed‐effect logistic regression method adjusted for the clustering effect of the multistage sampling design used in the FSNA survey. In the multivariate analysis we only included explanatory variables with *p* < 0.2 from the bivariate regression analysis. Multicollinearity of the variables was assessed by using the variance inflation factor (VIF > 10). In the multivariate analysis, we used a backward stepwise method to remove the nonsignificant variables (*p* > 0.05). To identify variables with interaction effects in the model, we assessed the significance (*p* > 0.05) of each interaction term one at a time in the basic model, but none of the interaction terms was conceptually meaningful. We found no confounding factors in our model after testing for a ≥10% change in the effect measure in the presence of another variable. We retained maternal stature and season of the survey in the final model although they were dropped during the backward stepwise process based on literature (Black et al., 2013; Harding et al., 2018). Akaike information criterion (AIC) and Bayesian information criterion (BIC) methods were used to assess for the goodness of fit of our model. Factors associated with WaSt were reported by adjusted odds ratio (aOR) at 95% confidence interval (CI). We set statistical significance at *p* < 0.05 in this analysis.

## 3 RESULTS

### 3.1 Characteristics of the study participants

**FIGURE 1:**
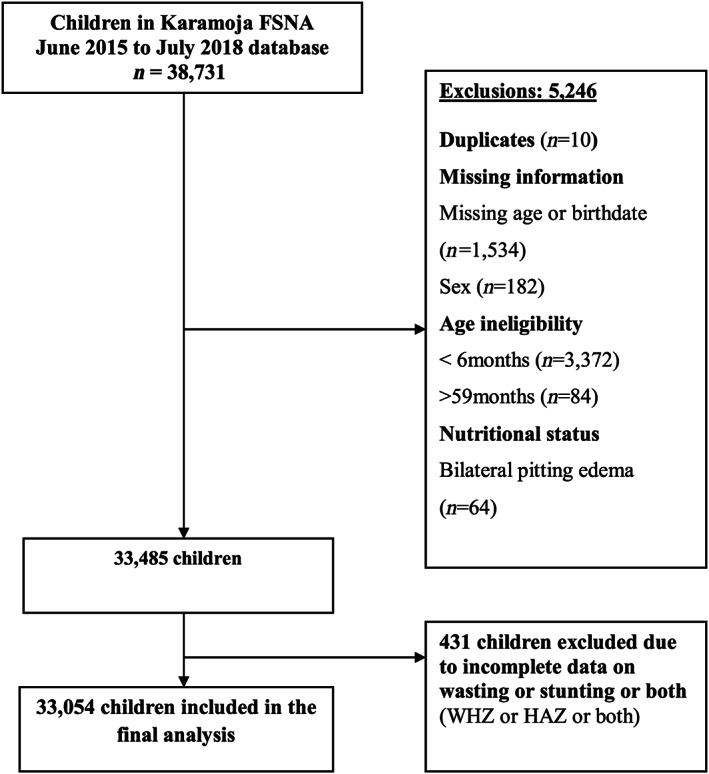
Flow chart showing participant selection among children 6–59 months in Karamoja (June 2015–July 2018 Food Security and Nutrition Assessment [FSNA]). HAZ, height for age z‐score; WHZ, weight for height z‐score

The study involved 33,054 children aged 6–59 months with available data on sex, wasting and stunting (Figure 1). About half (50.4%) of the children in the study sample were females. The median age of the children was 26 months (IQR: 15, 38), and about 44.3% were in the age group 6–23 months (Table 1). About 9.9% of the children had a diarrhoea episode and 26.0% had acute respiratory infection (ARI) in the 2 weeks before the survey. Nearly 59.6% of the children had caregivers with no formal education and about 27.0% of the caregivers were underweight (BMI < 18.5 kg/m^2^) (Table 1). About 47.9% resided in households that owned no livestock. Only about half (53.3%) of the households had an acceptable food consumption index. Almost two‐thirds (65.4%) of the children lived in households with no household toilet. Most of the households (62.9%) were surveyed during the hunger/lean season (Table 1).

**TABLE 1:** Characteristics of study participants, Karamoja, June 2015–July 2018 Food Security and Nutrition Assessment (FSNA) (*N* = 33,054 unless stated)

Attributes	Frequency (*n*)	Percentage (%)
Child's sex		
Female	16,647	50.4
Male	16,407	49.6
Child's age (months)		
Median (IQR)	26 (15, 38)	
Male; median age	26 (15, 37)	
Female; median age	26 (15, 38)	
Age group; *n* (%)		
6–23	14,658	44.3
24–59	18,396	55.7
ARI in the last 2 weeks		
No	24,442	74.0
Yes	8,612	26.0
Diarrhoea in the last 2 week		
No	29,781	90.1
Yes	3,273	9.9
Malaria/fever in the last 2 weeks		
No	22,465	68.0
Yes	10,589	32.0
Maternal age (years)		
≥40	3,694	11.2
30–39	10,822	32.7
20–29	17,361	52.5
15–19	1,177	3.6
Maternal education (years)		
≥8	1,570	4.7
1–7	11,797	35.7
No education	19,687	59.6
Number of live‐births (*n* = 32,522)		
1–3	15,945	49.0
4–6	12,302	38.0
> = 7	4,275	13.0
Maternal BMI^a^ (*n* = 26,919)		
Normal (18.5–24.9 kg/m2)	18,841	70.0
Underweight (<18.5 kg/m2)	7,310	27.0
Overweight (≥ 25Kg/m2)	768	3.0
Maternal stature^a^ (cm) (*n* = 27,401)		
170/max	3,822	14.0
160–170	15,429	56.3
Min/160	8,150	29.7
Maternal MUAC^b^ (*n* = 32,215)		
Normal (MUAC ≥23 cm)	27,065	84.0
Low MUAC (MUAC <23 cm)	5,150	16.0
Household head sex (*n* = 32,606)		
Male	24,836	76.2
Female	7,770	23.8
Household head education (years)		
≥8	4,327	13.1
1–7	5,758	17.4
No education	22,969	69.5
Household wealth index		
Richest	6,604	20.0
Rich	6,572	19.9
Middle	6,532	19.8
Poorer	6,526	19.7
Poorest	6,820	20.6
Household food consumption scores		
Acceptable	17,608	53.3
Borderline	10,640	32.2
Poor	4,806	14.5
Household livestock ownership		
Yes	17,207	52.1
No	15,847	47.9
Household land access		
Yes	28,428	86.0
No	4,626	14.0
Presence of latrine		
Yes	11,426	34.6
No	21,628	65.4
Sources of drinking water^c^(*n* = 32,561)		
Improved water sources	28,513	87.6
Unimproved water sources	4,048	12.4
Season of survey		
Harvest	12,274	37.1
Hunger	20,780	62.9

Abbreviations: BMI, body mass index; IQR, interquartile range; MUAC, mid‐upper arm circumference.

^a^ For nonpregnant women.

^b^ For both pregnant and nonpregnant women

^c^ Improved water sources (water through piped/tap, protected well/spring and borehole fitted with a hand‐pump). Unimproved water sources (surface water, river, dam, runoff, rainwater collected in a tank and water from open well/spring).

### 3.2 Prevalence of undernutrition

**FIGURE 2:**
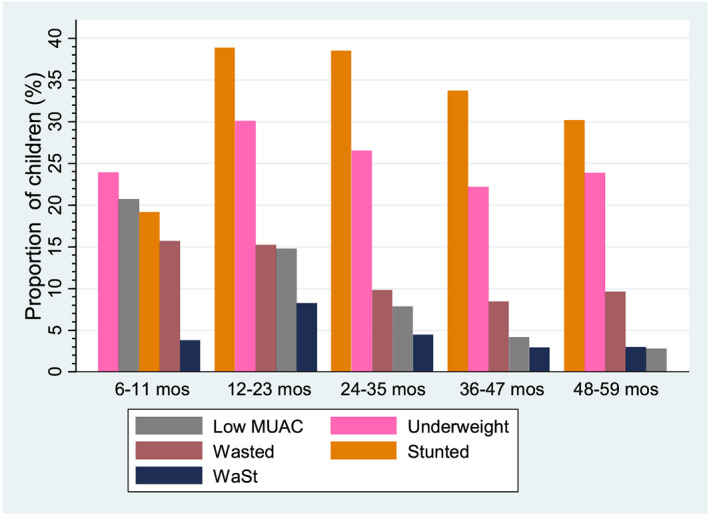
Proportion of undernutrition by age groups among children 6–59 months in Karamoja (June 2015–July 2018 Food Security and Nutrition Assessment [FSNA]). MUAC, mid‐upper arm circumference; WaSt, concurrent wasting and stunting

Of the 33,054 children in the sample, 10.6% (95% CI [10.2–11.0]) had low MUAC, 26.0% (95% CI [25.4–26.5]) were underweight, 12.0% (95% CI [11.6–12.5]) were wasted and 33.5% (95% CI [33.0–34.1]) were stunted. The prevalence of WaSt among the children in the sample was 5.0% (95% CI [4.6–5.3]) (Table 2). WaSt was more common among children aged <36 months than those aged >36 months. Stunting was the most prevalent form of undernutrition across the different age groups (Figure 2).

**TABLE 2:** Prevalence of undernutrition among children aged 6–59 months, Karamoja, June 2015–July 2018 Food Security and Nutrition Assessment (FSNA) (*N* = 33,054 unless stated)

Nutritional status	Frequency (*n*)	Percent (%)	95% CI
Low MUAC			
No deficit (MUAC ≥12.5)	29,473	89.4	89.1–89.7
Moderate (MUAC ≥11.5 to ≤12.5 cm)	2,811	8.5	8.2–8.8
Severe (MUAC <11.5 cm)	678	2.1	1.9–2.2
Overall low MUAC (MUAC <12.5 cm)	3,489	10.6	10.2–11.0
Underweight			
No deficit (WAZ ≥−2)	24,471	74.0	73.5–74.6
Moderate (WAZ ≥−3 to −2)	6,117	18.5	18.1–18.9
Severe (WAZ <−3)	2,466	7.5	7.1–7.8
Overall underweight (WAZ <−2)	8,583	26.0	25.4–26.5
Wasted			
No deficit (WHZ ≥−2)	29,076	88.0	87.5–88.5
Moderate (WHZ ≥−3 to −2)	3,035	9.0	8.8–9.6
Severe (WHZ <−3)	943	3.0	2.7–3.1
Overall wasted (WHZ <−2)	3,978	12.0	11.6–12.5
Stunted			
No deficit (HAZ ≥−2)	21,963	66.5	65.9–67.0
Moderate (HAZ ≥−3 to −2)	6,848	20.7	20.3–21.2
Severe (HAZ <−3)	4,243	12.8	12.4–21.2
Overall stunted (HAZ <−2)	11,091	33.5	33.0–34.1
Concurrently wasted and stunted			
WaSt	1,635	5.0	4.6–5.3

Abbreviations: CI, confidence interval; HAZ, height for age z‐score; MUAC, mid‐upper arm circumference; WaSt, concurrent wasting and stunting; WHZ, weight for height z‐score.

### 3.3 Factors associated with WaSt

Several child‐level, maternal, household, socioeconomic and seasonal variables were associated with WaSt at bivariate and multivariate analyses (Table 3). At multivariate analysis, male children had 1.79 (95% CI [1.60–2.00]) odds of being WaSt compared with females. Children aged 12–23 months (aOR = 2.25; 95% CI [1.85–2.74]) and 24–35 months (aOR = 1.15; 95% CI [0.95–1.40]) had increased odds of WaSt compared with children aged 6–11 months. Children aged 36–47 months (aOR = 0.65; 95% CI [0.50–0.84]) and 48–59 months (aOR = 0.71; 95% CI [0.54–0.93]) had lower odds of WaSt compared with children aged 6–11 months. Episodes of ARI (aOR = 1.30; 95% CI [1.15–1.48]) and diarrhoea (aOR = 1.25; 95% CI [1.06–1.48]) in the past two weeks before the survey increased the odds of WaSt. However, children who had malaria/fever 2 weeks before the survey were less likely to have WaSt (aOR = 0.83; 95% CI [0.73–0.96]). (Table 3).

**TABLE 3:** Bivariate and multivariate analyses of factors associated with concurrent wasting and stunting (WaSt) among children 6–59 months, Karamoja, June 2015–July 2018 Food Security and Nutrition Assessment (FSNA)

Attributes	Bivariate		Multivariate	
	cOR (95% CI)	*p* Value	aOR (95% CI)	*p* Value
Sex				
Female	1	—	1	—
Male	1.82 (1.63–2.03)	<0.001	1.79 (1.60–2.00)	<0.001
Age (months)				
6–11	1	—	1	—
12–23	2.28 (1.92–2.70)	<0.001	2.25 (1.85–2.74)	<0.001
24–35	1.18 (0.99–1.41)	0.071	1.15 (0.95–1.40)	0.157
36–47	0.76 (0.62–0.93)	0.008	0.65 (0.50–0.84)	0.001
48–59	0.78 (0.62–0.980	0.037	0.71 (0.54–0.93)	0.014
ARI in the last 2 weeks				
No	1	—	1	—
Yes	1.40 (1.25–1.560	<0.001	1.30 (1.15–1.48)	<0.001
Diarrhoea in the last 2 weeks				
No	1	—	1	—
Yes	1.22 (1.04–1.42)	0.015	1.25 (1.06–1.48)	0.009
Malaria/fever infection in the last 2 weeks				
No	1	—	1	—
Yes	0.80 (0.70–0.91)	0.01	0.83 (0.73–0.96)	0.010
Maternal age (years)				
≥40	1	—	—	—
30–39	0.98 (0.87–1.11)	0.788	—	—
20–29	0.71 (0.62–0.80)	<0.001	—	—
15–19	0.74 (0.54–1.00)	0.056	—	—
Maternal education (years)				
≥8	1	—	1	—
1–7	3.02 (2.03–4.48)	<0.001	2.12 (1.29–3.48)	0.003
No education	3.74 (2.56–5.48)	<0.001	2.08 (1.28–3.38)	0.003
Number of live‐births				
1–3	1	—	1	—
4–6	1.46 (1.31–1.62)	<0.001	1.51 (1.33–1.72)	<0.001
≥7	1.47 (1.26–1.720	<0.001	1.56 (1.32–1.85)	<0.001
Maternal BMI^a^ (kg/m^2^)				
Normal (18.5–24.9)	1	—	1	—
Underweight (<18.5)	2.06 (1.87–2.28)	<0.001	1.80 (1.58–2.04)	<0.001
Overweight (≥25)	0.48 (0.29–0.78)	0.003	0.62 (0.36–1.06)	0.078
Maternal height^a^ (cm)				
≥170	1	—	1	—
160–170	1.15 (0.94–1.42)	0.175	1.17 (0.94–1.47)	0.162
<160	1.44 (1.18–1.77)	<0.001	1.49 (1.22–1.83)	<0.001
Maternal MUAC^b^				
Normal (MUAC ≥23 cm)	1	—	1	—
Low MUAC (MUAC <23 cm)	2.02 (1.76–2.32)	<0.001	1.28 (1.09–1.51)	0.003

Household head gender				
Male	1			
Female	1.18 (1.05–1.33)	0.006	—	—
Household head education (years)				
≥8	1	—	1	—
1–7	2.00 (1.50–2.65)	<0.001	1.57 (1.21–2.04)	0.001
No education	2.42 (1.86–3.15)	<0.001	1.81 (1.37–2.41)	<0.001
Wealth index				
Richest	1	—	—	—
Rich	1.53 (1.32–1.79)	<0.001	1.29 (1.09–1.52)	0.003
Middle	1.98 (1.67–2.36)	<0.001	1.38 (1.12–1.70)	0.003
Poorer	2.02 (1.71–2.39)	<0.001	1.33 (1.10–1.60)	0.002
Poorest	1.92 (1.64–2.26)	<0.001	1.39 (1.17–1.65)	<0.001
Food consumption scores				
Acceptable	1	—	1	1
Borderline	1.27 (1.14–1.43)	<0.001	1.10 (0.97–1.24)	0.133
Poor	1.34 (1.16–1.55)	<0.001	1.13 (0.97–1.335)	0.124
Household livestock ownership				
Yes	1	—	—	—
No	1.35 (1.21–1.51)	<0.001	1.30 (1.13–1.49)	<0.001
Household access to land				
Yes	1	—	—	—
No	1.05 (0.90–1.23)	0.540	—	—
Presence of latrine				
Yes	1	—	—	—
No	1.27 (1.11–1.45)	<0.001	—	—
Sources of drinking water				
Improved water sources	1	—	—	—
Unimproved water sources	1.05 (0.90–1.22)	0.545	—	—
Season of survey				
Harvest	1	—	—	—
Hunger	1.17 (1.03–1.31)	0.012	1.14 (0.98–1.33)	0.084

Abbreviations: aOR, adjusted odds ratio; ARI, acute respiratory infection; CI, confidence interval; cOR, crude odds ratio; MUAC, mid‐upper arm circumference.

^a^ For nonpregnant women.

^b^ For both pregnant and nonpregnant women.

At multivariate analysis, caregivers with 4–6 live‐births (aOR = 1.51; 95% CI [1.33–1.72]) and ≥7 live‐births (aOR = 1.56; 95% CI [1.32–1.85]), maternal underweight (aOR = 1.80; 95% CI [1.58–2.04]), maternal short stature (aOR = 1.50; 95% CI [1.22–1.83]) and maternal low MUAC (aOR = 1.28; 95% CI [1.09–1.51]) were significantly associated with WaSt. The odds of WaSt was lower among children of overweight/obese caregivers (aOR = 0.62;95% CI [0.36–1.06]) compared with underweight caregivers though this was not statistically significant. Caregivers with no formal education (aOR = 2.12; 95% CI [1.30–3.48]), and caregivers with 1–7 years of education (aOR = 2.08; 95% CI [1.28–3.38]) were significantly associated with WaSt. Maternal age showed significant association with WaSt at bivariate analysis but dropped from the model at multivariate analysis (Table 3).

In the adjusted analysis, household heads with no formal education (aOR = 1.82; 95% CI [1.37–2.41]) and household heads who had 1–7 years of education (aOR = 1.57; 95% CI [1.21–2.04]) were significantly associated with WaSt. Households with the rich wealth index quintile had 1.29 (aOR = 1.29; 95% CI [1.09–1.52]) odds of WaSt compared with the richest households. Children living in households with middle (aOR = 1.38; 95% CI [1.12–1.70]), poorer (aOR = 1.33; 95% CI [1.11–1.60]) and poorest (aOR = 1.39; 95% CI [1.18–1.65]) wealth index quintiles had higher odds of WaSt compared with those that lived in the richest households. The odds of WaSt were 1.30 times higher among children living in households without livestock compared with those that had livestock (aOR = 1.30; 95% CI [1.13–1.49]). Household food consumption scores, latrine ownership, household access to land for cultivation and seasons were not significantly associated with WaSt (Table 3).

## 4 DISCUSSION

We found that the child's sex, age, history of ARI, diarrhoea and malaria/fever episode in the last weeks were associated with WaSt at the individual level. Male children were 1.79 times more likely to experience WaSt compared with females. This finding corroborates earlier analyses showing that males were more susceptible to WaSt (Garenne et al., 2019; Khara et al., 2018; Myatt et al., 2018; Schoenbuchner et al., 2019). This is contrary to previous studies where female children were seven times more likely to suffer multiple anthropometric deficits than males (Fentahun et al., 2016). This difference could be explained by the difference in the case definition of multiple anthropometric deficits as a composite index for all forms of undernutrition versus children without any deficit compared with the current study that considered two deficits.

The higher odds of WaSt in males compared with females seen in our study emphasize the high vulnerability of males to wasting and stunting compared with females (Harding et al., 2018; Khara et al., 2018; Martorell & Young, 2012; Poda, Hsu, & Chao, 2017). A meta‐analysis showed that males have a 1.18 times higher risk of stunting compared with females, but the sex difference in stunting was not related to socioeconomic status in sub‐Saharan Africa (Wamani, Astrom, Peterson, Tumwine, & Tylleskar, 2007).

The reason for the sex difference in undernutrition remains unclear. Males are more likely to be stunted and to experience poor complementary feeding practices compared with females (Bork & Diallo, 2017). In another study, there were no sex differences in malnutrition and feeding patterns (Condo, Gage, Mock, Rice, & Greiner, 2014). Garenne et al. (2019), hypothesized that stunting in boys is associated with sex‐specific hormones such as testosterone, luteinzing hormone and follicle‐stimulation hormone (FSH). For instance, FSH disappears after 6 months in boys affects stunting. On the other hand, FSH stays at high levels in girls until 3–4 years (Kuiri‐Hanninen, Sankilampi, & Dunkel, 2014). Further, Garenne et al. (2019) have demonstrated that prior to age 30 months, males were 1.6 times more likely to be WaSt than females, but, the sex difference disappeared after age 30 months. We recommend further studies to examine the sex differential in WaSt assessments, to broaden our understanding of the aetiology of WaSt.

The child's age was an important correlate of WaSt in the current study. The odds of WaSt decreased with an increase in age. Previous studies also reported that WaSt prevalence peaks at 12–23 months and then declines after 36 months (Garenne et al., 2019; Myatt et al., 2018). Other studies have reported that prevalence of wasting decreases as age increases while stunting prevalence increases with age (Boah, Azupogo, Amporfro, & Abada, 2019; Martorell & Young, 2012; Poda et al., 2017). The existing literature shows that growth faltering in children starts at a much earlier age up to 24 months and with some bumps after 24 months (Victora, de Onis, Hallal, Blossner, & Shrimpton, 2010). These findings highlight the need to target younger children particularly those <36 months for infant and young child feeding, growth monitoring and promotion interventions.

In our analysis, recent morbidity of ARI and diarrhoea infections in the past 2 weeks prior to the survey increased the odds of WaSt in children. Our results are in line with previous studies, which showed that children with multiple anthropometric deficits were more likely to experience any morbidity including ARI (Fentahun et al., 2016). The relationship between undernutrition and infections is synergistic; undernutrition is a risk factor for infection and vice versa (Scrimshaw & SanGiovanni, 1997). It is well established that diarrhoea is associated with undernutrition (Guerrant, Schorling, McAuliffe, & de Souza, 1992). An analysis of nine longitudinal datasets showed that the odds of stunting increased by 1.13 for every five episodes of diarrhoea and 25% of all stunting was attributed to five previous episodes of diarrhoea (Checkley et al., 2008). In another analysis, diarrhoea episode was attributed to weight loss, and a small but measurable decrease in linear growth over the long term (Richard et al., 2013).

Furthermore, we found that children with malaria/fever had a reduced risk of having WaSt. Evidence on the relationship between malaria and undernutrition remains inconclusive as some studies have found no influence of malaria on the occurrence of wasting and stunting (Das et al., 2018). Studies in Ethiopia and Uganda found malaria/fever infection to be strongly associated with stunting and wasting (Gari, Loha, Deressa, Solomon, & Lindtjorn, 2018; Wamani, Astrom, Peterson, Tumwine, & Tylleskar, 2006). A recent analysis showed that children with concurrent wasting, stunting and underweight had 1.34 odds of experiencing prolonged days of illness (Hondru et al., 2019). These findings show that children with WaSt are at high risk of morbidity. Therefore, prompt treatment and prevention of infections through integrated approaches to improve caregivers' care practices, household socioeconomic status and environmental health are recommended (Dodos et al., 2018).

Children of caregivers who had over four live‐births were more likely to experience WaSt compared with children who had caregivers with less than three live‐births. WaSt was strongly associated with low maternal BMI, stature and low MUAC in this analysis. These findings resonate with a previous analysis that shows that low maternal BMI and stature were associated with wasting and stunting (Martorell & Young, 2012). In another study, children whose caregivers had low MUAC (MUAC <210 mm) were three times likely to be MAM than children with well‐nourished caregivers (MUAC ≥210 mm) (Bahya‐Batinda, Dramaix‐Wilmet, & Donnen, 2018). There is evidence that foetal growth is restricted during pregnancy in women of short stature and low BMI (Black et al., 2013). Pregnant women with low BMI, short stature and low MUAC had a high risk of maternal, neonatal and child death and low birth weight (Black et al., 2013; Christian et al., 2008; Nyamasege et al., 2018; Ververs et al., 2013). Poor maternal nutritional status could be an indication of poor household feeding practices.

In the present study, we also found that the children whose caregivers were overweight were 0.38 times less likely to be WaSt, although this effect was not statistically significant. These findings imply that in protracted food insecure contexts there could be some overweight/obese caregivers, probably food secure with high socioeconomic status who could afford to feed their children adequately. Maternal and child undernutrition are interconnected and have long‐term and intergenerational consequences (Victora et al., 2008). Wasting and stunting co‐exist at birth and persist concurrently over time in some children (Wells et al., 2019). Therefore, interventions targeted at improving maternal and child nutrition, health and well‐being should focus on the first 1,000 days (i.e., from conception until 24 months) of a child's life (Schwarzenberg & Georgieff, 2018).

Contrary to previous studies, household characteristics such as the presence of latrine, safe drinking water and food consumption scores were not associated with WaSt. Children who lived in households with improved latrine and source of drinking water were protected from stunting and wasting in other studies (Agho, Akombi, Ferdous, Mbugua, & Kamara, 2019; Harding et al., 2018; Poda et al., 2017; van Cooten, Bilal, Gebremedhin, & Spigt, 2019; Wamani et al., 2006). Furthermore, some reports have shown an association between household food consumption scores and stunting (Bukusuba, Kaaya, & Atukwase, 2017; Saaka & Osman, 2013). Future analysis is recommended on the relationship between household water and sanitation (water, sanitation and hygiene [WASH]); food security seasons and WaSt to broaden our understanding of causality of WaSt.

Household heads and caregivers with no or low level of formal education were more likely to have children with WaSt than those with secondary education. Additionally, children belonging to middle, poor and poorest wealth households had increased risk of WaSt. These results were confirmatory of previous studies indicating that these socioeconomic factors were associated with wasting and stunting (Boah et al., 2019; Harding et al., 2018; Poda et al., 2017; Wamani et al., 2006). Parental education especially the mother's education and household wealth index are important socioeconomic correlates of undernutrition; these characteristics may influence child feeding and care practices and the enabling environment for child growth and development (Akombi et al., 2017; Wamani et al., 2006).

The risk of WaSt was higher among children in a household without livestock. A previous report showed that a 10‐fold increase in household livestock ownership had a significant association with lower stunting prevalence in Ethiopia and Uganda, but not in Kenya (Mosites et al., 2015). Karamoja is predominantly pastoralist and agro‐pastoralist, and household livestock ownership is indicative of wealth, resilience and livelihood. Apart from being used as food, livestock rearing is a financial livelihood that can serve as credit, asset‐based insurance and agricultural labour for land traction (Behnke & Arasio, 2019). Actions for improving livestock productivity in Karamoja should be a priority because of the beneficial effects on child nutrition and well‐being.

Our analysis showed no significant association between seasons and WaSt. However, earlier analyses showed that food security seasons were predictors for wasting and stunting in a Gambian longitudinal study (Schoenbuchner et al., 2019) and a cross‐sectional study conducted in Somalia (Kinyoki et al., 2016). Karamoja is chronically food insecure and prone to recurrent drought and sporadic flooding. These harsh living conditions make children susceptible to malnutrition and infectious diseases. In such a setting, persistent stunting will be more common than persistent wasting (Richard et al., 2012). The actual burden of wasting may not be revealed in cross‐sectional data; this may be because wasting is an acute condition and wasted children die or recover over a short period. Wasting is appropriately measured by incidence (Isanaka, Boundy, Grais, Myatt, & Briend, 2016), whereas stunting, a chronic condition, can be measured by prevalence data. Because WaSt consists of wasting and stunting, a relationship with food security may be better explained by a longitudinal study rather than the cross‐sectional data used in this study. Longitudinal studies on WaSt and food security seasons may provide better understanding and evidence for decision making.

Although our understanding of the epidemiology and aetiology of WaSt is still emerging, extant reports assert that wasting and stunting share similar causal pathways (Briend et al., 2015; Harding et al., 2018; Khara & Dolan, 2014; Kinyoki et al., 2016). Results on factors associated with WaSt reported in this study may not differ from factors attributable to wasting and stunting in the literature (Khara & Dolan, 2014). Our results add to the call for integrated approaches to prevent and treat simultaneous wasting and stunting in children (Bergeron & Castleman, 2012). Future programmatic and policy decision making should consider interventions that promote linear and ponderal growth at the same time (Harding et al., 2018). In the context of high undernutrition burden, children with WaSt should be recognized as a public health priority group for treatment given the heightened risk of death associated with WaSt.

### 4.1 Strengths and limitations

Previous analyses reported on factors associated with the different forms of nutrition outcomes. To the best of our knowledge this is the first study to assess factors associated with WaSt, a useful composite indicator for wasting and stunting (Myatt et al., 2018). However, the interpretation of our study should consider some limitations commonly associated with a secondary dataset. The likelihood of random error because of a fixed sample size may have been offset by the large sample used in the present analysis. We may have introduced selection bias by excluding about 21% of the children with incomplete data, missing data, and implausible anthropometric data. Nevertheless, children who were excluded from the study had similar sex distribution patterns, though they were younger.

Missing data and erroneous anthropometric measurements by field enumerators were probable sources of information bias in the FSNA dataset. However, information bias was likely to be minimal due to the regular and standardized training of field enumerators and the use of standard operating procedures for sampling and data collection during each round of the survey.

Given that the present study was based on existing dataset, we were limited to explore the effects of the variables found in the FSNA dataset. We could not easily infer causality of WaSt because the dataset was cross‐sectional. Moreover, wasting has a shorter duration and is more seasonally variable compared with stunting. Therefore the true burden of wasting may be under‐reported in prevalence data (Khara & Dolan, 2014). However, the results presented in this report could be useful baseline information for future studies on factors associated with WaSt and provide useful evidence for decision making on interventions for the prevention and treatment of WaSt.

## 5 CONCLUSION

WaSt was significantly associated with the child's age, sex, ARI and diarrhoea episodes especially among children whose caregivers had low BMI, short stature, low MUAC, ≥4 live‐births and low socioeconomic status. Cost‐effective interventions aimed at preventing and treating childhood illnesses and improving prenatal and maternal nutrition, women empowerment and socioeconomic status will be beneficial for early childhood nutrition and well‐being. Existing programmes and interventions such as community‐based management of acute malnutrition (CMAM) designed solely for the treatment of wasting, food and micronutrient supplementation, infant and young child feeding programme, social safety net, and hygiene and sanitation promotion for stunting prevention should be adapted to address both conditions, especially in high burden settings like Karamoja. There is a need to identify and test how integrated packages of nutrition‐sensitive and nutrition‐specific interventions can be targeted at reducing wasting and stunting in children. Future prospective studies on the risk factors of WaSt are needed to improve our understanding of the causality of WaSt.

## CONFLICTS OF INTEREST

The authors declare no conflicts of interest.

## CONTRIBUTIONS

GAOO‐A, HW, MM, AB, JNK and CASK conceptualized and designed the research study. GAOO‐A, HW, RA, JN, JO, YK, JNK, MM, AB and CASK analysed and interpreted the data. GAOO‐A drafted the initial manuscript. GAOO‐A, HW, JN, JO, YK, JNK, EM, RA, MM, AB and CASK reviewed and finalized the manuscript.
